# “After my husband’s circumcision, I know that I am safe from diseases”: Women’s Attitudes and Risk Perceptions Towards Male Circumcision in Iringa, Tanzania

**DOI:** 10.1371/journal.pone.0074391

**Published:** 2013-08-29

**Authors:** Erica H. Layer, S. Wilson Beckham, Lilian Mgeni, Catherine Shembilu, Romani B. Momburi, Caitlin E. Kennedy

**Affiliations:** 1 Department of International Health, Johns Hopkins Bloomberg School of Public Health, Baltimore, Maryland, United States of America; 2 Department of Psychiatry, Muhimbili University of Health and Allied Sciences, Dar es Salaam, Tanzania; 3 Primary Health Care Institute, Iringa, Tanzania; University of Ottawa, Canada

## Abstract

While male circumcision reduces the risk of female-to-male HIV transmission and certain sexually transmitted infections (STIs), there is little evidence that circumcision provides women with direct protection against HIV. This study used qualitative methods to assess women’s perceptions of male circumcision in Iringa, Tanzania. Women in this study had strong preferences for circumcised men because of the low risk perception of HIV with circumcised men, social norms favoring circumcised men, and perceived increased sexual desirability of circumcised men. The health benefits of male circumcision were generally overstated; many respondents falsely believed that women are also directly protected against HIV and that the risk of *all* STIs is greatly reduced or eliminated in circumcised men. Efforts to engage women about the risks and limitations of male circumcision, in addition to the benefits, should be expanded so that women can accurately assess their risk of HIV or STIs during sexual intercourse with circumcised men.

## Introduction

Three randomized controlled trials (RCTs) and multiple observational studies have shown that male circumcision reduces the risk of female to male HIV transmission by up to 60% [1–4]. Circumcised men are also at decreased risk of genital ulcer disease (GUD), herpes simplex virus type 2 (HSV-2), *trichomonas vaginalis* and high-risk human papillomavirus (HR–HPV) [5]. Due to these health benefits, the World Health Organization recommends male circumcision scale-up in countries with high HIV prevalence and low rates of male circumcision, and several sub-Saharan African countries are rolling out large-scale programs [6,7]

While male circumcision partially reduces the risk of HIV and other STIs in men, the direct health benefits of male circumcision for women are less clear. A meta-analysis of 19 studies, including one RCT and six longitudinal studies, found little evidence that male circumcision directly reduces risk of HIV acquisition in women [8]. As male circumcision programs expand, HIV prevalence is expected to decrease in circumcised men. Several mathematical models of male circumcision scale-up have predicted a long-term population-level benefit in which HIV prevalence will also decrease in women as a result of decreased HIV prevalence among circumcised men [9–11]. However, it is estimated that the full effect of these indirect benefits could take decades to be realized [12].

Until the population-level benefits of male circumcision are realized for women, there are specific situations that could put women at increased risk of HIV infection. First, female partners of circumcised men may be at increased risk of HIV if sex is initiated before the recommended six week post-circumcision abstinence period [13]. A recent study in Kenya has documented high levels of early initiation of sexual intercourse following circumcision [14], and a mathematical model predicts that moderate levels of early sexual intercourse following circumcision could lead to more new HIV infections in women in the first year than would otherwise have occurred in the absence of male circumcision scale-up [15].

Another threat to women in male circumcision programs is risk compensation. Risk compensation, or behavioral disinhibition, is the increase in risk behaviors as a result of a reduction in perceived risk [16,17]. Both men and women may change their behavior based on their risk perception which could lead to increased risk of HIV. For example, if circumcised men have more sexual partners or reduce condom usage, or if women are more willing to have unprotected sex with circumcised men because they believe they are at less risk of disease, the protective effects of male circumcision could be offset [18]. Little risk compensation has been observed among men during the three RCTs in South Africa, Uganda and Kenya [1–3,19,20]. However, intensive behavioral counseling, along with free and unlimited provision of condoms, may have contributed to a decrease in risk behaviors among all trial participants which may not be applicable to programmatic settings.

Little is known about the impact of male circumcision on risk behaviors of men or women in circumcision programs outside of RCT settings. If men or women believe that male circumcision provides more protection than it actually affords, they may be more likely to engage in risk compensation, which would have the most adverse effect on women. A recent study by Dushoff and colleagues predicts that even small amounts of risk compensation in male circumcision programs, which would still likely decrease HIV incidence in men, could lead to an increase in HIV incidence in women [12,21]. 

In areas where male circumcision is being scaled up, it is important that women not only have a comprehensive understanding of the benefits, but also the limitations of male circumcision, including that circumcision does not signify an HIV-negative status, that HIV transmission to men and women is still possible when a man is circumcised, and that women do not receive direct protection if a circumcised man is HIV-positive. This knowledge could help women to more accurately assess their own risk of HIV. Several recently published articles suggest a poor understanding of the limitations of male circumcision. For example, in Kisumu, Kenya, 26% of women and 19% of men agreed that condom use is less necessary now that male circumcision is available [22]. Additionally, Lundsby and colleagues found that many circumcised men in a qualitative study in Zambia believe they have a “slim” chance of contracting HIV [23]. A further study in three sub-Saharan African countries found that the benefits of male circumcision were exaggerated by many respondents [24].

In addition to understanding how women perceive the health benefits and risks of male circumcision, understanding wider social norms emerging in parallel to scale-up can be useful in monitoring any changes in women’s sexual preference associated with men’s circumcision status. Understanding these changes in social norms and perceptions can help programs to tailor communication messages that resonate with current social norms.

This qualitative study examines women’s perceptions of male circumcision in Iringa, Tanzania – a region experiencing rapid male circumcision scale-up. We assess women’s understanding of male circumcision as a partially protective HIV prevention strategy for men as well as the social value women place on male circumcision.

## Methods

### Study site

This study was conducted in the Iringa region of Tanzania. Iringa is located approximately 500 kilometers southwest of the commercial capital, Dar es Salaam. At the time of the study, the Iringa Region consisted of six districts, with a total population of 1.5 million people [25]. HIV prevalence among adults in Iringa is the highest in the country at 16%, which is more than 2.5 times the national average of 5.7% [26]. Male circumcision is not commonly practiced in Iringa; rates of male circumcision in this region are among the lowest in the country at 30%, compared to 67% nationally [26]. However, there do not appear to be barriers to male circumcision acceptance. A national situational analysis on male circumcision in Tanzania found broad acceptance for male circumcision. In this study, 76% of non-circumcised males said that they would be circumcised if services were available. In addition, 93% and 98% of non-circumcised and circumcised males, respectively, and 89% of females supported circumcision for their sons [27]. 

Iringa Region is currently experiencing scale-up of male circumcision services. Circumcision is offered through government health services to men ages 10-49 free of charge. The program focuses on the availability of free male circumcision services by trained health care providers and the HIV preventive benefits of male circumcision [28]. Various channels are used to increase demand for services including radio announcements, billboards and experiential media [29]. The Tanzanian government has set targets to circumcise 2.8 million men countrywide by 2015, including 264,990 in the Iringa Region [30].

### Participant recruitment

In order to understand women’s attitudes and risk perceptions towards male circumcision, we conducted in-depth interviews (IDIs) with both HIV-negative and HIV-positive married women whose husbands were circumcised during the previous year. To triangulate across methods, we also conducted focus group discussions (FGDs) with married and unmarried women [31]. Recruitment relied on self-reported HIV status and husbands’ circumcision status. Participants were purposively sampled from women’s groups, HIV support groups and health centers in urban and rural areas of Iringa Region [32]. Snowball sampling was also used to identify eligible interview participants. Study participants and leaders of relevant organizations were given business cards and asked to share them with women who might be eligible to participate. When data collectors recruited women for the study, they specified that they were not associated with the male circumcision program in Iringa in order to reduce social desirability bias.

### Data collection and analysis

We conducted IDIs with 18 HIV-negative and 15 HIV-positive married women whose husbands had been circumcised in the previous year. Because interviews with married women covered sensitive topics such as sexual behavior before and after their husbands’ circumcisions, we conducted follow-up interviews whenever possible in order to build rapport with the study participants and gain a more in-depth understanding of their experiences. Of the 33 interview participants, 16 were interviewed once, 14 were interviewed twice, and three were interviewed three times; this led to a total of 53 IDIs (Table 1). IDIs lasted between 20 and 90 minutes. Six FGDs with 53 participants were conducted with married (n=3) and unmarried (n=3) women. FGDs consisted of between seven and 12 participants and lasted from 65 to 90 minutes. Participants were compensated with 3,000 Tanzanian Shillings (~US$ 2) at the end of each IDI or FGD for their time and transport. This amount was chosen because it is generally enough for a meal and local transport fees, but is not high enough to be considered undue inducement.

**Table 1:** Overview of IDIs and FGDs.

**In-depth interviews with married women whose husbands were circumcised in the previous year**				
	One interview	Two interviews	Three interviews	Total
HIV-negative	7	9	2	18 participants
HIV-positive	9	5	1	15 participants
**Focus group discussions**				
Married women				3 groups
Unmarried women				3 groups

Semi-structured guides were used for both interviews and focus group discussions. In-depth interview guides focused on the woman’s understanding of the benefits and limitations of male circumcision, the decision-making process that she and her partner went through prior to her partner’s circumcision (including the woman’s role in her partner’s decision), her experience shortly after the circumcision and any changes in the couple’s relationship dynamics. Focus group discussion guides included questions about women’s general understanding of male circumcision, social norms around male circumcision, information received about male circumcision and ways in which women obtain information, and women’s perceptions about the benefits and limitations of male circumcision. Questions were open-ended and probing was used to explore additional topics that arose throughout the interview. At the end of each IDI or FGD, data collectors provided information to participants to clarify any misconceptions that arose throughout the conversation. Participants were also invited to ask any questions about the content of the interviews. Data collectors answered general questions to the best of their ability. In addition, if data collectors felt that participants had any harmful misconceptions about male circumcision or questions that were beyond the scope of what the data collector could answer, participants were referred to a local health facility for further counseling and/or support.

All IDIs and FGDs were conducted in Kiswahili and digitally recorded with the permission of the respondents. Data collectors took extensive notes during each interview and wrote a memo on the same day identifying main points from the session. All data were transcribed in Kiswahili and fully translated into English within one week. Weekly meetings were held in which the study team discussed findings from the interviews and the lead author read all transcripts as soon as they were available to provide feedback to the data collectors so that emerging themes could be explored in more depth. Memos were also written by the lead author to capture meaning from each interview and to summarize main themes from the data [33]. The study team determined that data saturation was reached since no new information was being revealed [34,35]. A codebook was developed by the study team based on emerging themes from the data and all transcripts were then coded using Atlas. ti version 6.2. Following data coding, matrices were developed to compare findings between in-depth interviews with married women whose husbands were recently circumcised and focus group discussions with married women and unmarried women. Findings were compared across matrices to determine if themes differed by marital status or data collection method. Consensus on final themes was reached by the study team through multiple discussions and reading of interview transcripts to confirm results.

### Ethics Statement

IDIs and FGDs were conducted by four university-educated Tanzanian women who were trained in an intensive two week course on qualitative research theory and methods, interviewing techniques, and human subjects research ethics. Since no other identifying information was collected for the study, asking individuals to disclose their full names by providing written consent would decrease their anonymity, so oral informed consent was obtained from all participants prior to enrollment. The research assistants read a consent form to all participants prior to enrollment. If the participant consented, the research assistant signed two copies of the form; one was given to the participant and one was kept with the Principal Investigator. This research, including the decision to obtain oral informed consent, was approved by the Johns Hopkins Bloomberg School of Public Health Institutional Review Board and the Tanzania National Institute for Medical Research.

## Results

Women in this study discussed their perceptions, understanding and emerging social norms around circumcision. Three broad themes emerged: (1) social pressures for men to be circumcised; (2) low risk perception of sexual intercourse with circumcised men; and (3) increased desirability of circumcised men. We found no major differences in understanding of the benefits and risk of male circumcision or attitudes towards circumcised men between married and unmarried women, HIV-positive and HIV-negative women, or between women in IDIs and FGDs; therefore, we present results for the entire study population together.

### Social pressure for men to be circumcised

Participants reported a very strong social pressure for men to be circumcised. Uncircumcised men were described as being dirty, uneducated and “out of fashion.” One woman described uncircumcised men as follows:

*Uncircumcised men are perceived as being uneducated. The community doesn’t like men who know about the benefits of male circumcision but don’t act on that knowledge and get circumcised.* –Unmarried woman, FGD

Another recurrent theme was the shame associated with being uncircumcised. One woman discussed how embarrassed she would be if someone found out that her husband was uncircumcised:

*I felt bad because my husband was not circumcised. I told him 'Will you go for circumcision? It might happen that you suddenly get sick and people might volunteer to come and take care of you. If they find you that way [uncircumcised], in my heart, I will feel ashamed...'* –Married woman, HIV-positive, IDI

Women repeatedly discussed embarrassment associated with men being uncircumcised. Many participants explained that men were uncomfortable to bathe or urinate near other men for fear of being teased:

*Uncircumcised men feel ashamed in front of their fellow men. For example, when an uncircumcised man urinates in front of his friends, they might see him and start to make fun of him and say "Eh, you’re not circumcised! You have not removed your 'mzula' (cap)," and they laugh at him too.* –Married woman, HIV-negative, IDI

Similarly, married women reported being very embarrassed for other women to discover that their husbands were not circumcised

*If women in our village know that your husband is not circumcised, they will laugh at you and say ‘What do you have? Your husband is not even circumcised. He still has a 'mkonosweta' [sweater sleeve].’ That woman will feel shame when her friends laugh at her.* – Married woman, HIV-negative, IDI

### Low risk perception of HIV through sexual intercourse with circumcised men

#### Men have a greatly reduced risk of HIV

A common theme discussed by participants was the belief that uncircumcised men were at very high risk of disease. Circumcised men were described as having reduced levels of disease which was one of their most desirable attributes.

*I was worried that he was not circumcised because it’s easy for uncircumcised men to get HIV. I had so many questions in my head like 'What if he goes outside marriage? Since he is not circumcised, he might get infected.' So when he went for circumcision my heart was filled with joy*. –Married woman, HIV-negative, IDI

Similarly, women mentioned that messages about the health benefits of male circumcision made them feel safer when their husbands were circumcised

*I heard from the radio and brochures that a real man is one who is circumcised because he reduces most of his risk of disease. So after my husband’s circumcision, I know that I am safe from diseases*. –Married woman, HIV-negative, IDI

Many women explained that while men were at a reduced risk of disease, they could still acquire HIV when circumcised

*Yes, a man can still get HIV even if he is circumcised. If he has sex with a woman who is already infected with HIV, he can also be infected even if he is circumcised*. –Married woman, HIV-negative, IDI

However, participants also reported that it is “not easy to get infected” or that risk of disease is eliminated in circumcised men

*It reduces the rate of HIV transmission in a sense that if a man is circumcised and he is not HIV-positive then he cannot become infected* … *For example, I am HIV positive. If I have sex with an uncircumcised man and I refuse to use a condom, I will surely transmit the disease to him. But if he is circumcised he will not get HIV*. –Married woman, HIV-positive, IDI

#### Women are directly protected from HIV

Furthermore, many participants believed that women were directly protected against HIV as long as a man is circumcised, even if a man was HIV-positive. Many noted that they heard that male circumcision reduces the risk of HIV, and assumed that this applied to both men and women. Others described how women are protected from HIV if a man is circumcised:

*Even circumcised men who are HIV-positive can have sex with women and the women will not get the infection easily.* – Married woman, FGD

Some participants also stated that the only way women could protect themselves against disease was for their sexual partners to be circumcised.

*Personally, I don’t think a woman can protect herself from diseases if her husband is not circumcised; it’s too difficult. A man is the one who cleans himself, just like I clean myself. So I don’t think there is any safe way for a woman to prevent herself from diseases if her husband is not circumcised. The best way for a woman to be safe is for her husband to be circumcised*. –Married woman, HIV-negative, IDI

#### Protection against STIs

Reduction of STIs was mentioned as one of the most important benefits of male circumcision. When asked about which STIs were reduced, almost all women mentioned syphilis and gonorrhea. Furthermore, many women reported that the risk of *all* STIs was either reduced or eliminated once a man is circumcised. One woman responded that male circumcision reduces the risk of “all the sexually transmitted infections like syphilis and gonorrhea.”

### Circumcised men are more sexually desirable than uncircumcised men

#### Women prefer circumcised men as sexual partners

Many unmarried participants said they would refuse to have sex with an uncircumcised man. Uncircumcised men were perceived as being dirty and having diseases:

*One day, when I was coming back from school I saw a girl standing next to a boy. They were arguing and the girl said 'you’re not even circumcised. Go and be circumcised first, then you can come back to me.' … I think the girl knew that because the boy was not circumcised, he was at very high risk of having HIV and STIs*. –Unmarried woman, FGD

In contrast, participants discussed how women are much more willing to have sex with a circumcised man due to his elevated social status

*Circumcised men feel proud in front of other people because everyone knows that circumcision is good. Circumcised men can find sexual partners easily. But if a woman finds that a man is not circumcised, she will not agree to be his lover*. –Married woman, HIV-negative, IDI

Some women discussed how uncircumcised men may be unable to find sexual partners because of the shame associated with not being circumcised. It was suggested multiple times that uncircumcised men sometimes resort to female sex workers or, in extreme cases, rape because other women refuse sex with uncircumcised men. According to one woman:

*It is very difficult for uncircumcised men to find women willing to have sex with them. Therefore, such a person [uncircumcised man] can go to a local bar where he knows he will find cheap women [female sex workers] and they will have sex in dark places where she cannot tell he is uncircumcised. But circumcised men can find women easily at any time; not in dark places*. –Married woman, FGD

A few married women threatened to leave their husbands if they did not get circumcised, while others told stories of female friends who initiated sexual partnerships with circumcised men after their uncircumcised husbands refused to be circumcised:

*There was a woman who was telling her friends 'Every day I tell my [uncircumcised] husband that when we have sex, he does not satisfy me. But he just ignores me; he does nothing. I tell him to go for circumcision but he refuses.' So this woman looked for another man who was circumcised. She still lives with her husband ... but she is having an affair*. –Married woman, HIV-negative, IDI

#### Circumcision increases sexual pleasure for women

Overwhelmingly, women reported increased sexual pleasure with circumcised men compared to uncircumcised men. Women discussed how they did not enjoy sexual intercourse with uncircumcised men because they often felt pain and the man needed to pull back his foreskin during sexual intercourse. One woman described her experience before her husband was circumcised:

*Before he was circumcised his penis was very large because of the foreskin. But a circumcised man's penis is different from an uncircumcised man’ s penis. Before he was circumcised, my husband needed to hold his foreskin with his hand in order for us to have sex. We both had pain then. The foreskin seemed to be an obstacle when we had sex*. –Married woman, HIV-negative, IDI

In contrast, many women reported no pain or discomfort with circumcised men and often discussed the ease with which men could have sexual intercourse once their foreskin was removed.

*Male circumcision makes a woman enjoy sex because during sexual intercourse with a circumcised man, even the power of the man having sex increases. Before my husband was circumcised, when we had sex, the foreskin was going up and back frequently, causing him to feel pain. Since he has been circumcised, I enjoy sex much more and my husband does not feel pain*. –Married woman, HIV-negative, IDI

### Male circumcision messaging

The male circumcision program in Iringa utilizes a variety of media channels to create demand for male circumcision by emphasizing the benefits of male circumcision [27]. Participants in our study were very aware of these messages and mentioned that they heard about the benefits of male circumcision from the radio, billboards, loudspeakers on cars and posters at health centers. The way that they spoke about circumcision also indicated familiarity with the campaign messages. For example, the phrase *dondosha mkonosweta* (translated in English as “drop your sweatersleeve” referring to a man being circumcised) was widely used on billboards, pamphlets and signs throughout Iringa. In a majority of interviews, women referred to circumcision as *dondosha mkonosweta* instead of the traditional Swahili word for male circumcision, *tohara ya mwanaume*. In addition, billboards and signs promoting male circumcision in Iringa displayed a handsome, muscular man wearing a t-shirt with the phrase *mwanaume zaidi 60%* (translated in English as “60% more man”) and many participants made reference to this during interviews. Referring to this campaign, one married participant said “My husband is now 60% more of a man [since he has been circumcised].”

All participants said they heard about the benefits of male circumcision and announcements urging men to be circumcised. As one woman described it:

*On the radio they were saying it is important for a man to be circumcised so as to reduce the risk of acquiring infections like gonorrhea, syphilis, and HIV – that was what they were saying*. –Married woman, FGD

Another participant said that radio advertisements help men feel safe:

*He [circumcised husband] feels safer because even when he is having sex he is at peace … Circumcised men feel safer because even in the radio they mentioned that [circumcised men] will not get HIV.* – Married woman, HIV-positive, IDI

### Conceptual Framework

**Figure 1:**
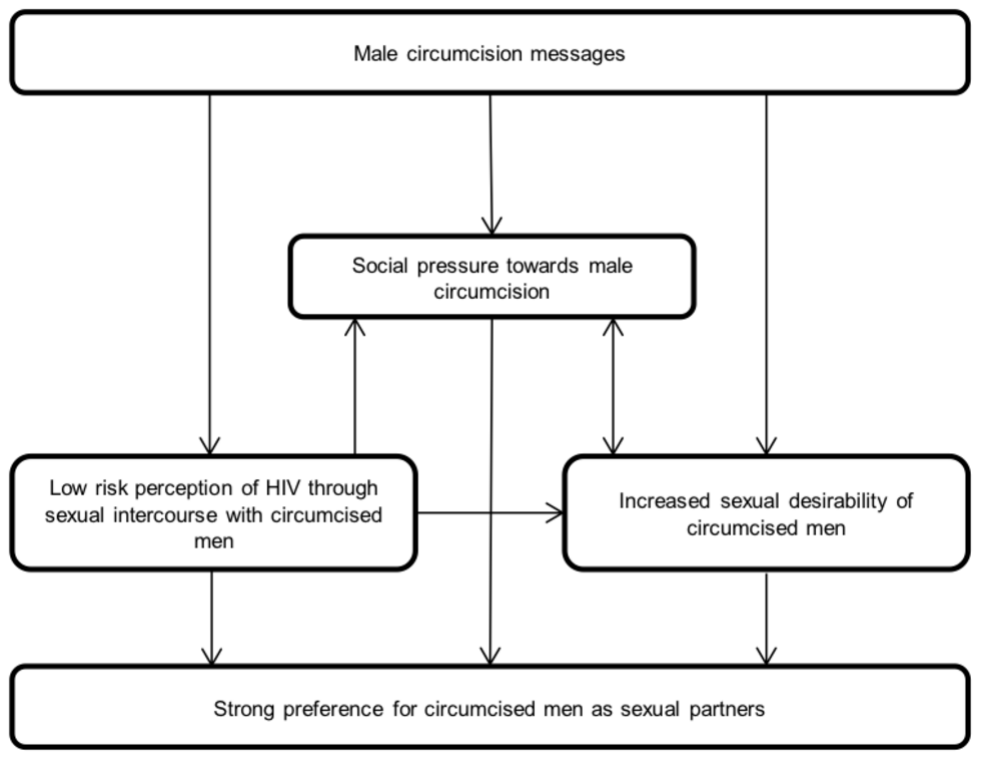
Conceptual framework of factors affecting women’s perceptions of male circumcision.

Figure 1 describes the way in which the themes presented above are mutually reinforcing and lead to women’s strong preference for circumcised men as sexual partners. The most commonly discussed benefit of male circumcision by women in this study was the reduced risk of HIV and STIs. Messages promoting male circumcision for its health benefits were widely known and the main reason that women mentioned for men to be circumcised. The perception that circumcised men are healthy, strong and disease free influenced social norms and increased sexual desirability of circumcised men. There is strong social pressure for men to be circumcised in Iringa and women commonly discussed the shame felt by uncircumcised men and female partners of uncircumcised men. These social norms appear to be shaped by the messages promoting male circumcision, by the belief that male circumcision greatly reduced or eliminates the risk of HIV and STIs, and by the increased desirability of circumcised men. Similarly, the increased sexual desirability of circumcised men seems to be driven by a combination of the media campaigns portraying circumcised men as strong, attractive and healthy as well as the perceived health benefits and strong social pressure towards circumcision. Each of these themes independently contributes to women’s strong preferences for circumcised men as sexual partners in Iringa.

## Discussion

Our findings indicate that women in Iringa, Tanzania strongly prefer circumcised men because of the low risk perception of HIV with circumcised men, social norms favoring circumcised men, and perceived increased sexual desirability of circumcised men. The combination of these emerging social norms and ideas about increased sexual pleasure with circumcised men, coupled with an exaggerated belief in the health benefits, suggests that male circumcision is increasingly being seen as not only a desirable trait, but one that is required of men who wish to find female sexual partners. In addition, women’s lack of understanding of limitations of male circumcision in this study may lead to increased risk-taking by women who believe they are more protected against HIV than they actually are. 

Women in this study discussed many social norms around male circumcision which lead to a preference for circumcised men as sexual partners. First, women commonly reported shame or embarrassment associated with uncircumcised men. Not only do other men laugh at uncircumcised men, but women also make fun of them and their female partners. The social desirability of circumcision led some married women in this study to insist that their husbands get circumcised. Additionally, many unmarried women in this study reported that they would refuse sexual intercourse with uncircumcised men. These findings have several implications. First, demand creation programs can capitalize on the increased social acceptance of circumcised men in order to increase uptake of circumcision. However, too strong an emphasis on circumcision may lead to stigma. In this study, women suggested that some uncircumcised men may actually be so ashamed of their circumcision status, or so humiliated by the perceived ridicule and rejection from their peers and potential sex partners, that they resort to sex with female sex workers or even rape. Although it is not clear whether this type of extreme behavior was a rumor or whether it had actually happened in Iringa, other studies have suggested that male circumcision programs may lead to stigma. Eaton and Kalichman predicted that male circumcision scale-up could lead to stigmatization of uncircumcised men, as lack of circumcision could be perceived as a marker for HIV-positive status [36]. Our findings suggest that many uncircumcised men are stigmatized for many social reasons which include, but are not limited to, HIV status and disease risk. Understanding emerging social norms around male circumcision can help programs to tailor messages to minimize any negative impact on uncircumcised men.

In addition to the increased social pressure for men to be circumcised, women overwhelmingly reported increased sexual pleasure with circumcised men. Other previously published articles assessing women’s sexual satisfaction with circumcised men have found marginally increased sexual satisfaction following circumcision [22,37]. Married women with recently circumcised partners in our study almost unanimously stated that their sexual pleasure increased following circumcision, which was mainly attributable to decreased pain and men not needing to manually hold back their foreskin during sex.

While women have many social reasons for preferring circumcised men, our participants tended to overstate the health benefits for circumcised men and their female partners. Women were aware that male circumcision is only partially protective, but many women thought that a circumcised man’s risk of acquiring HIV is very low and that it is “not easy” or “difficult” for circumcised men to acquire diseases. Similar findings have been reported in Zambia [38]. While male circumcision does reduce the risk of female-to-male HIV acquisition, some men will still acquire HIV after circumcision despite the partial protection offered by male circumcision. Additionally, HIV-positive men are routinely circumcised as long as clinical conditions do not indicate otherwise [6]. It is important that women understand that a man’s circumcision status does not signify a negative HIV status and that the risk of acquiring HIV, while reduced, still exists. 

Many women in this study felt that they received direct protection against HIV if their partners were circumcised. Some women assumed that the 60% reduced risk of HIV infection in men applied to women as well. Messages about the 60% reduced risk of HIV should be careful to articulate that the directly reduced risk of HIV applies to men but not women, that circumcised men may be HIV-positive, and that women do not receive protection against HIV if their circumcised partners are HIV-positive. 

The belief that male circumcision reduces or eliminates the risk of all STIs was very common. The most frequently mentioned STIs were syphilis and gonorrhea, likely because these are the most familiar STIs in this setting [39]. A qualitative study in Zambia reported a similar misconception among circumcised men [23,39]. However, there is no biomedical evidence of any reduced risk of these infections. Male circumcision has been shown to reduce the risk of GUD, HSV-2, *trichomonas vaginalis* and HR–HPV in men and HR–HPV, bacterial vaginosis, *trichomonas vaginalis* and GUD in women [5,40]. While the reduction of these STIs is significant and should be promoted, it is also important that women understand that circumcision does not affect the risk of all STIs.

We found little evidence that women fully understood the limitations of male circumcision or the potential health risks specific to women. Because of the potential increase in risk for women associated with modest levels of risk compensation or early resumption of sexual activity following circumcision, multiple articles have stressed the importance of behavior change campaigns accompanying male circumcision programs [12,41,42]. Increasing the number of messages that promote decreased risk behavior and provide women with balanced information about male circumcision could help women to more accurately assess their risk of HIV or STIs during sexual intercourse with circumcised men.

This study has several limitations. First, we conducted multiple in-depth interviews with married women whose husbands were previously circumcised, but only conducted three focus group discussions with unmarried women. Unmarried women, who are less likely to have regular sexual partners, may have different perceptions of male circumcision, and in-depth interviews with these women as opposed to a group setting may have yielded different results. Additionally, in this paper we explore women’s attitudes and risk perceptions towards male circumcision but do not measure the extent to which risk compensation is or is not occurring among women. A further quantitative study assessing the correlation between women’s understanding of male circumcision and risk behaviors could be useful. Finally, since this study was conducted in one region of Tanzania, these findings may not be generalizable to other male circumcision programs throughout sub-Saharan Africa. Despite these limitations, this study provides unique insight into women’s attitudes and risk perception of male circumcision in Tanzania which may be useful to other male circumcision programs.

## Conclusion

In conclusion, our findings indicate that male circumcision is becoming socially normative in Iringa, Tanzania, and that women strongly prefer circumcised men. Male circumcision scale-up efforts in Iringa have generated demand among men and cultivating strong support for male circumcision among women. Promotion of the social benefits of male circumcision including social acceptance of circumcised men and increased sexual pleasure among women could be effective approaches to generate even more demand for services. 

Women’s strong support for male circumcision was partially derived from the many perceived health benefits to both men and women. While women understand that male circumcision reduces the risk of HIV and other STIs, misconceptions such as direct protection against HIV and reduced risk of *all* STIs were common. The social desirability of circumcised men, in combination with women’s exaggerated belief in the health benefits of male circumcision, may create an environment in which women are more likely to engage in unprotected sexual intercourse with circumcised men. In order for women to make informed decisions about their health, programs should increase gender-specific messages to women which highlight both the benefits and limitations of male circumcision.
